# Modulation of Motoneuronal Activity With Sleep-Wake States and Motoneuronal Gene Expression Vary With Circadian Rest-Activity Cycle

**DOI:** 10.3389/fnint.2018.00032

**Published:** 2018-08-07

**Authors:** Kate B. Herr, Graziella L. Mann, Leszek Kubin

**Affiliations:** Department of Biomedical Sciences, School of Veterinary Medicine, University of Pennsylvania, Philadelphia, PA, United States

**Keywords:** affymetrix microarrays, circadian rhythm, hypoglossal motoneurons, inferior olive, rest-activity cycle, sleep apne, tongue

## Abstract

In both nocturnal and diurnal mammals, sleep and wake states differentially aggregate during the rest and active phases of circadian cycle. Closely associated with this rhythm are prominent changes in motor activity. Here, we quantified the magnitudes of electromyographic activity (EMG) measured separately during different sleep-wake states across the rest-activity cycle, thereby separating amplitude measurements from the known dependance of the timing of wake and sleep on the phase of circadian rest-activity cycle. In seven rats chronically instrumented for electroencephalogram and EMG monitoring, nuchal and lingual muscle EMGs were measured as a commonly used postural output in behavioral sleep studies and as a cranial motor output with potential clinical relevance in obstructive sleep apnea (OSA) syndrome, respectively. We found that, for both motor outputs, EMG measured during wake episodes was significantly higher during the active phase, than during the rest phase, of circadian cycle. The corresponding patterns observed during slow-wave sleep (SWS) and rapid eye movement sleep (REMS) were different. During SWS, lingual EMG was very low and did not differ between the rest and active phase, whereas nuchal EMG had pattern similar to that during wakefulness. During REMS, lingual EMG was, paradoxically, higher during the rest phase due to increased twitching activity, whereas nuchal EMG was very low throughout the rest and active periods (postural atonia). In the follow-up comparison of differences in transcript levels in tissue samples obtained from the medullary hypoglossal motor nucleus and inferior olive (IO) at rest onset and active period onset conducted using microarrays, we identified significant differences for multiple transcripts representing the core members of the molecular circadian clock and other genes important for the regulation of cell metabolism and activity (up to *n* = 130 at *p* < 0.001). Collectively, our data indicate that activity of motoneurons is regulated to optimally align it with the rest-activity cycle, with the process possibly involving transcriptional mechanisms at the motoneuronal level. Our data also suggest that OSA patients may be relatively better protected against sleep-related upper airway obstructions during REMS episodes generated during the rest phase, than during active phase, of the circadian cycle.

## Introduction

Hypoglossal (XII) motoneurons innervate the muscles of the tongue. In obstructive sleep apnea (OSA) patients, contraction of these muscles is required to protect the pharyngeal airway from collapse and ensure effective breathing (Remmers et al., 1978; Jordan et al., 2010; White and Younes, 2012; Kubin, 2016). This protective action often fails during sleep which results in sleep loss and fragmentation. This, in turn, leads to sleepiness, as well as sleep bouts occurring outside of the natural rest phase of circadian cycle (e.g., naps during the day).

The important role of the tongue as an airway-protecting muscle in OSA makes the mechanisms of its sleep/wake and circadian-dependent controls clinically important. To date, due to the especially high relevance of sleep-wake changes in upper airway muscle tone for the maintenance of upper airway patency during sleep, most studies in both humans and animal models focused on the central neural control of upper airway muscles during the rest phase of circadian cycle (e.g., Hendricks et al., 1993; Katz and White, 2003; Lu et al., 2005; Smith et al., 2007; Bastedo et al., 2009; Nicholas et al., 2012). Consequently, any differences in the sleep-wake modulation of upper airway motor output over the entire 24-h period of the circadian cycle have been little investigated. In our recent study in behaving rats, we reported that the magnitude of lingual electromyographic activity (EMG) measured during wakefulness is higher during the active period of circadian cycle (night in rats) than during the adjacent rest periods (Kubin and Mann, 2018). Additional day-night differences in the control of lingual EMG may occur during rapid eye movement sleep (REMS; Rukhadze et al., 2012). Other reports indicate that the propensity for central apneic episodes varies in rats with the phase of circadian cycle (Fink et al., 2014), and that time of the day (ToD) affects respiratory chemosensitivity, the frequency and duration of respiratory events, and propensity for upper airway collapse during slow-wave sleep (SWS) in human OSA subjects (El-Chami et al., 2014, 2015). Accordingly, there are compelling reasons to investigate whether the effects of sleep-wake states on muscle tone vary with the phase of the rest-activity cycle.

The first goal of our present study was to quantify the time course of sleep-wake changes in lingual and nuchal (postural) EMGs across the entire circadian cycle. Since we found highly significant, potentially clinically relevant and distinctly expressed in different stages of sleep effects, we followed with a complementary screen for genes that exhibit differential expression in the XII motor nucleus between the day/rest and night/active portions of the circadian cycle. We compared these transcriptional changes to those occurring in the medullary inferior olive (IO) region (a site also involved in motor control but located upstream from motoneurons), and with the mouse brainstem data derived from a publicly available database specifically focused on circadian oscillations (CircaDB, 2018). We determined that a subset of transcripts that are a part of the core molecular clock exhibit highly significant day-night differences in the XII nucleus. This suggests that circadian variations observed at the motor output, while undoubtedly controlled by the neural and humoral influences emanating from the master circadian clock located in the hypothalamus, are also reinforced by local molecular changes occurring at the motoneuronal level.

Preliminary data from a part of the study have been published (Kubin et al., 2015).

## Materials and Methods

### Animals

We used 21 adult male Sprague-Dawley rats whose body weight at the time of study entry was 306–450 g. Of the 21 rats, seven of the nine rats subjected to chronic instrumentation, and 12 out of 13 rats designated for brain regional gene expression studies with microarrays yielded complete results included in the present report; data from the remaining three rats were excluded due to technical problems. All experimental procedures followed the National Institutes of Health (USA) *Guide for the Care and Use of Laboratory Animals* and were reviewed and approved by the Institutional Animal Care and Use Committee of the University of Pennsylvania (protocol number: 804875).

### Animal Instrumentation for Chronic Recording of Electroencephalographic (EEG) and Electromyographic (EMG) Activity

After acclimatization to our housing environment, under aseptic conditions, rats were subjected to surgical anesthesia that was initially induced with ketamine (70 mg/kg, i.m.) and xylazine (10 mg/kg, i.m.) and then maintained with isoflurane (1.0%–1.5%). They were instrumented for chronic wired monitoring of lingual EMG, nuchal EMG and cortical EEG, as described previously (Lu et al., 2005). In short, two multi-strand, Teflon-coated except 1 mm at the tip wires were placed in the muscles of the tongue using the approach described in detail elsewhere (Lu et al., 2005). The wires were tunneled subcutaneously and connected to a 9-pin connector attached with dental acrylic to the animal’s skull. Another two Teflon-coated wires were sutured onto the dorsal neck muscles and their other ends were connected to the head connector. Three wires were attached with stainless steel screws to burr holes drilled in the skull; of these, two served as EEG leads and the third as electrical reference. To ensure unambiguous identification of the location of recording sites in the tongue, lingual EMGs were acquired in monopolar configuration relative to a reference point on the skull (Lu and Kubin, 2009). Ultimately, one of the two lingual EMG signals obtained from each animal was selected for analysis based on its location near the base of the tongue and the quality and stability of the signal over multiple recording sessions.

After instrumentation surgery, to avoid damage to the implants, the animals were housed singly in adjacent standard cages, and with environment-enriching items provided. Water and standard rat chow were available at all times. Light timers were set to maintain a fixed 12:12 h light-dark cycle (lights on at 7:00 am). Starting on day 6–8 after instrumentation, each animal was accustomed to handling and experimental conditions through a series of habituation sessions. These included days and nights when the animal was placed in the recording chamber for progressively longer periods of time up to 24 h without being connected to the recording cable. This was followed by at least two overnight recording sessions conducted solely for the purpose of habituation and evaluation of the quality and stability of the recorded signals. All electrical signals were continuously acquired using the data acquisition hardware and Spike-2 software (Cambridge Electronic Design, Cambridge, England). The A/D conversion rate was 100 Hz for EEG and 1000 Hz for all EMGs. Concurrently, animal locomotor activity in the horizontal plane was monitored based on interruptions of infrared beams spaced at 2.5 cm along the long axis of the cage (MicroMax, AccuScan Instruments, Columbus, OH, USA). Individual beam breaks were counted which yielded a locomotor activity measure termed “total activity.” The counts were summed over successive 3 h-long intervals that were aligned with the intervals in which sleep-wake behavior and EMGs were quantified.

The habituation sessions were completed 18–48 days after instrumentation, after which one approximately 26 h-long recording designated for complete analysis was conducted starting at 2:00 pm and continuing until 4:00 pm on the next day. Out of this recording, data collected during the first 2 h were discarded as potentially confounded by a disruption of behavior caused by the recording hook-up procedure.

### Verification of the Location of Recording Sites in the Tongue

**Figure 1:**
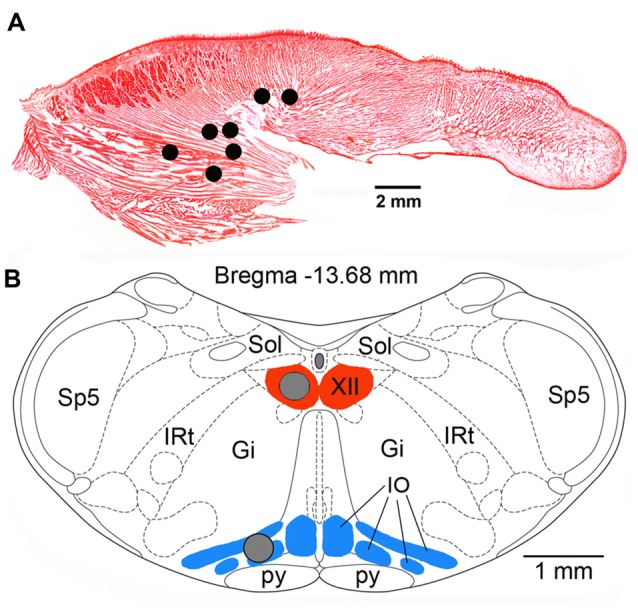
**(A)** Lingual electromyographic activity (EMG) recording sites in the seven rats used in this study superimposed onto a Neutral red-stained, sagittal cross-section of the rat tongue. All sites were localized within the posterior portion of the tongue either within the region mainly occupied by the genioglossal muscle fibers or at sites where such fibers are intermixed with intrinsic muscles. **(B)** Locations of the tissue micropunches (gray circles) harvested from the hypoglossal (XII) nucleus and the inferior olive (IO) superimposed onto a standard cross-section of the lower medulla from a level 13.68 mm caudal to Bregma of a rat brain atlas (Paxinos and Watson, 2007). Abbreviations: Gi, gigantocellular reticular region; IRt, intermediate reticular region; Sol, nucleus of the solitary tract; Sp5, spinal trigeminal sensory nucleus; py, pyramidal tract.

At the conclusion of all recording procedures, the animals were deeply anesthetized with pentobarbital (100 mg/kg, i.p.) and transcardially perfused with phosphate-buffered saline (PBS) followed by 4% paraformaldehyde in PBS. To determine the locations and integrity of the recording wires implanted in the tongue, with the implants kept intact, the tongue was gradually sliced in parasagittal plane with a scalpel blade under microscopic control until the bare tips of the recording wires were uncovered. Once the tips were found, their electrical connection to the head connector was verified, and their locations were then re-drawn onto a standard sagittal cross-section of a rat tongue (Figure 1A).

### Processing of Tissue Samples and Hybridization on Microarrays

All rats used in this part of the study were housed in our vivarium in groups of two or three under the normal 12:12 light-dark conditions for at least 2 weeks. Batches of two or three rats at a time were then deeply anesthetized with isoflurane (4%–5%), decapitated, and the lower brainstem was rapidly extracted and placed on the chuck of a vibratome (Ted Pella, Redding, CA, USA) in a cold medium. Two adjacent transverse slices, 500 μm-thick each, were obtained from the level just caudal to the opening of the central canal. Under a dissecting microscope, two 400 μm tissue punches were extracted and stored frozen at −80°C in separate PCR tubes. One punch was taken from the center of the XII nucleus, and the other from the inferior olive (IO) at the site of its widest cross-section (Figure 1B). Samples from 6 rats were collected around 6:45 pm, i.e., 15 min prior to the active period onset, and from another six rats around 7:15 am, i.e., 15 min after the rest period onset. RNA was isolated from individual samples using RNAeasy Mini columns (Qiagen, Hilden, Germany) and all subsequent procedures, including RNA reverse-transcription, cDNA amplification, labeling, hybridization onto microarrays, microarray reading, and initial processing were conducted at the Molecular Profiling Facility of the University of Pennsylvania. The Affymetrix GeneChip Rat Exon 1.0 ST was used for hybridization following procedures similar to those described elsewhere (Buechel et al., 2011; Willmann et al., 2011).

### Data Analysis

The states of wakefulness, non-rapid eye movement (NREM; or slow-wave sleep—SWS) and REMS were scored in successive 10 s epochs based on visual inspection of the signals assisted by epoch-by-epoch power spectral analysis (Somnologica-3, Medcare, Buffalo, NY, USA), as described previously (Lu et al., 2005; Lu and Kubin, 2009). Subsequently, root mean squares of lingual and nuchal EMG above the noise level were calculated for each scoring epoch and the epochs were sorted by behavioral state and the time of their occurrence within the recording session. The mean EMG levels were then measured separately for each state within the successive 3 h-long intervals starting at 4:00 pm and ending at 4:00 pm on the next day. To allow for comparison of EMG levels across different animals, mean EMG levels within different sleep-wake states occurring during successive 3 h-long intervals were normalized by the mean obtained during wakefulness at 4–7 am, i.e., when wake-related EMG is typically the highest.

Statistical analysis of sleep-wake amounts, EMG levels and locomotor activity included testing all data sets for normality and equal variance. This was followed by one-way repeated measures analysis of variance (ANOVA), with the examined factor being the sequential number of the 3 h-long analysis interval within the recording session. Statistical significance levels were corrected for multiple comparisons using the Holm-Sidak method (SigmaPlot v. 12.5, Systat Software, San Jose, CA, USA). When warranted, ANOVA was followed by paired Student’s *t*-tests, with the two-tailed *p* levels of 0.05 regarded statistically significant.

Microarray data were statistically analyzed using PARTEK Genomic Suite v. 6.5 (Partek Inc., St. Louis, MO, USA) with corrections for background noise, unspecific binding and using GC-RMA algorithms designed for the Affymetrics Gene Chips. Three factors were included in the subsequent ANOVA. The main factor of interest was the ToD; samples were collected either at active period onset, or at rest period onset, which yielded two levels. For brevity, in the text and the associated data worksheets, these two times are being referred to as “N” or “A-N” (for the active period/night onset) and “D” or “R-D” (for the rest period/day onset), respectively. The additional two factors were: the region from which the samples were obtained (XII nucleus or IO; two levels), and the data set number (to account for the fact that samples were collected and pre-processed in batches of two or three rats at a time on three different dates separated by several weeks; accordingly three distinct data set numbers were assigned to different samples in the entire data set). An Excel worksheet is associated with this report in which the first tab contains the entire unprocessed data set sorted by the significance level for ToD effect in the XII nucleus. Additional tabs (Tabs 2–10) contain the following: (2) a list of explanations for the column titles for Tabs 1 and 3–9; (3) data subset limited to the known transcripts only sorted by ToD significance level in the XII nucleus; (4) data subset limited to the known transcripts for which the significance levels for ToD effect for either the XII nucleus or IO were less than 0.001; (5) data subset limited to the known transcripts for which the significance levels for ToD effect for both the XII nucleus and IO were below 0.001; (6) data subset limited to the known transcripts for which the significance levels for ToD effect for the XII nucleus, but not for IO, were less than 0.001; (7) data subset limited to the known transcripts for which the significance levels for ToD effect for the IO, but not XII nucleus, were less than 0.001; (8) data subset limited to the known transcripts for which the significance levels for ToD effect for either the XII nucleus of IO were less than 0.01; (9) data subset for the key central molecular clock-related genes regardless of their ToD significance level in our samples; (10) data subset comprising all unknown genes in our data set, regardless of their ToD significance level sorted by the significance level for ToD in the XII nucleus. Tabs 1 and 3 through 10 include original microarray readings for each sample and transcript, individual transcript-by-transcript statistical comparisons for all factors, as well as the “stepped up” *p* values obtained using microarray-specific correction for multiple comparisons. The last tab (Tab 11) provides a tabulated comparison of the known transcripts in our study for which statistical significance of ToD effect in the XII nucleus was less than 0.001 and/or those that are a part of the core circadian oscillator to the measures of circadian cycling for the same genes, as assessed by microarray technology in the whole mouse brainstem and published in the “CIRCA” database (CircaDB, 2018). This database was selected for comparison as anatomically closest to our focus on two specific brainstem nuclei and as one providing an unbiased assessment of changes occurring with the circadian period.

## Results

### Sleep-Wake and Locomotor Activity Patterns Across the Rest-Activity Cycle

Under the normal light-dark conditions, the timing of sleep and wake is determined by the master circadian pacemaker located in the suprachiasmatic nucleus of the hypothalamus. This basic rhythm entrains secondary pacemakers distributed in the brain and peripheral organs through both neural and humoral pathways, and is expressed in the rest activity rhythms of locomotion, attention, EEG pattern, metabolism and multiple other physiologic functions (e.g., Trachsel et al., 1986; Grasing and Szeto, 1992; Frank and Heller, 1997; Beersma and Gordijn, 2007; Granados-Fuentes et al., 2012; Li et al., 2012; Borbély et al., 2016).

**Figure 2:**
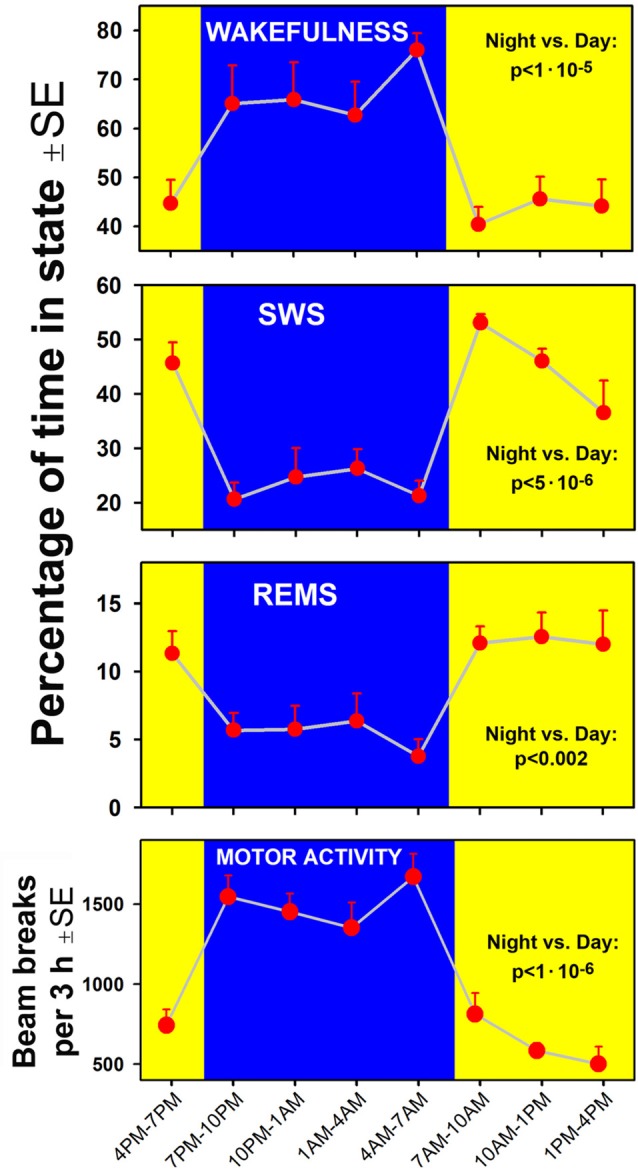
Time course of changes in the percentage amounts of wakefulness, slow-wave sleep (SWS) and rapid eye movement sleep (REMS; three top graphs) and locomotor activity (bottom) in successive 3 h-long intervals covering one full circadian cycle in the seven rats of the present study. Comparisons of the corresponding variables calculated for the entire night/active period (7:00 pm–7:00 am, blue areas) and the day/rest period (yellow areas) yielded statistically significant differences at *p* values from 0.002 to 1:10^6^.

A clear circadian pattern of sleep-wake and locomotor activity was present in the seven rats of our study. Figure 2 shows the average percentage amounts of sleep-wake states and motor activity tracked in successive 3 h-long intervals across the rest and active periods of circadian cycle. As expected, wakefulness occupied 60%–70% of time during the night/active period and, conversely, the combined amounts of SWS and REMS occupied about 60% of the day/rest period. For each measure shown in Figure 2, a comparison between the night and day epochs yielded highly significant statistical differences.

### Magnitude of Sleep-Wake Changes in Lingual and Nuchal EMGs Across the Rest-Activity Cycle

**Figure 3:**
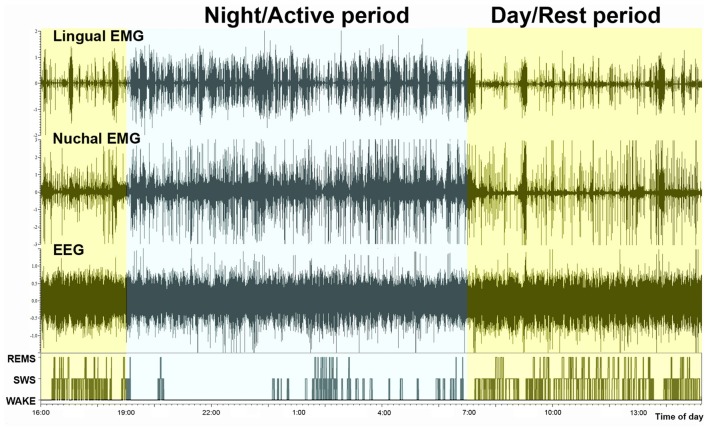
Continuous recording of lingual EMG, nuchal EMG and cortical EEG covering nearly 24 h. The bottom trace shows the time course of changes in sleep-wake states, with the typically reduced amount of sleep during the night/active period (7:00 pm–7:00 am) and increased occurrence of both SWS and REMS bouts during the day/rest period. It is of note that the active period (night) is characterized not only by an increased frequency and durations of wakefulness bouts but also by their particularly high amplitudes in both motor outputs.

**Figure 4:**
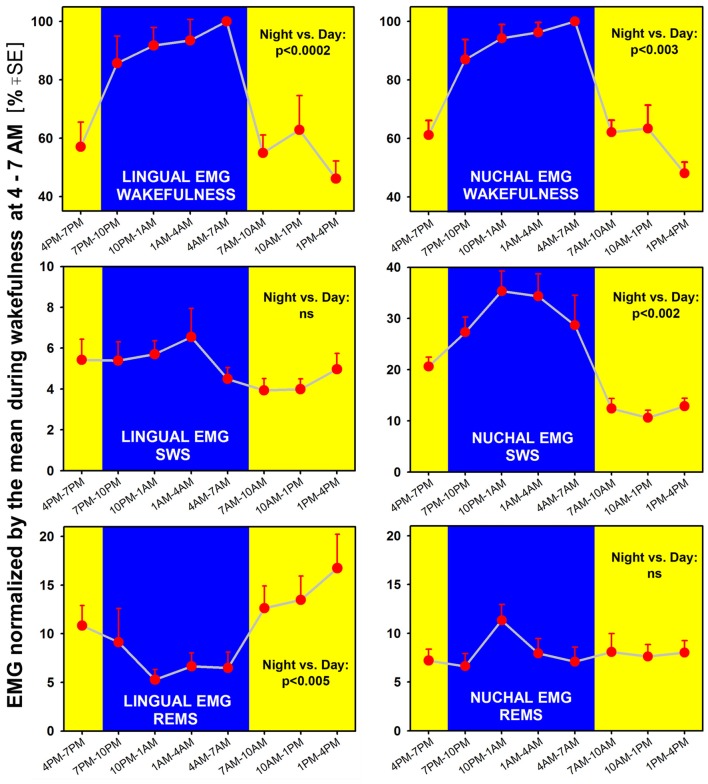
When measured separately in different sleep-wake states across a 24 h period, lingual (left) and nuchal (right) average EMGs show both state- and motor output-characteristic patterns. Both EMGs are significantly higher during wakefulness when measured during the night/active period than during the day/rest period, with *p* values for the day-night differences at 0.0002–0.003 (top graphs). Notably, lingual EMG in REMS is higher during the rest period than during the active period (bottom-left). In contrast, during SWS (middle graphs), lingual EMG is very low (4%–6% of the mean reference activity) and does not exhibit any significant day-night variation, whereas nuchal EMG is significantly higher during the night/active period than during the day/rest period (consistent with nuchal EMG pattern during wakefulness). Thus, during REMS, the lingual and nuchal EMG patterns are opposite to those during SWS because it is lingual, not nuchal, EMG that exhibits a significant day-night difference. All measurements are based on 24 h-long recordings in seven rats.

Examination of typical raw EMG records over the entire circadian cycle reveals that, in addition to the well-established day-night variation of the amounts of wakefulness, sleep states, durations of individual sleep and wake epochs, and motor activity, EMG amplitudes during individual bouts of wakefulness are higher during the active period than during the rest period of the circadian cycle. This is evident in the example shown in Figure 3. However, to evaluate this effect quantitatively requires that EMG activities be measured separately within the boundaries of distinct sleep-wake states. Towards this end, we used our earlier established and validated method of EMG quantification based on automated calculation of root mean squares of raw EMG in successive 10 s-long scoring epochs (Lu et al., 2005; Lu and Kubin, 2009). Figure 4 shows the outcomes from such an analysis for lingual and nuchal EMGs.

We found that both lingual and nuchal EMGs were significantly higher during wakefulness when measured during the night/active period than during the day/rest period (Figure 4; top graphs). Indeed, the peak magnitude of spontaneous EMG was about twice lower during the rest period than during the active period. While this difference was likely driven, in part, by the day-night differences in motivated behaviors, such as feeding and locomotion, a quantification of this difference has not been attempted in the past and is a necessary prerequisite for more detailed studies of the underlying mechanisms.

Interestingly, EMG quantified during sleep states did not fully follow the pattern seen during wakefulness and differed between the lingual and nuchal output. During SWS (Figure 4; middle graphs), lingual EMG was very low (4%–6% of the mean activity during wakefulness at 4:00–7:00 am) and did not exhibit any significant day-night variation. This likely reflected the fact that lingual muscles become atonic, or nearly atonic, on transition from wakefulness to SWS (Lu and Kubin, 2009; Rukhadze et al., 2011). In contrast, nuchal EMG measured during SWS was significantly higher during the night/active period than during the day/rest period. As such, it had a pattern of day-night difference like that for nuchal EMG during wakefulness, but the day-night difference was even larger. Specifically, SWS nuchal EMG during the night/active period was about 35% of the reference wakefulness level, whereas SWS-related nuchal EMG during the day/rest period was at about 10% of the reference wake-related activity.

During REMS, the lingual and nuchal EMGs had patterns opposite to those during SWS. Specifically, the lingual, but not nuchal, EMG exhibited a significant day-night difference and, notably, was lower during the night/active period than during the day/rest period. The elevation of lingual EMG during REMS when compared to SWS was causes by the differences in magnitude and frequency of lingual twitches (see “Time Course of Lingual and Nuchal EMG Changes During Transitions from SWS Sleep to REMS Across the Rest-Activity Cycle” section). On the other hand, the REMS-related nuchal EMG was very low throughout the 24 h period, indicating that the characteristic of REMS postural atonia was maintained during both the rest and active phases of circadian cycle.

### Time Course of Lingual and Nuchal EMG Changes During Transitions From SWS Sleep to REMS Across the Rest-Activity Cycle

**Figure 5:**
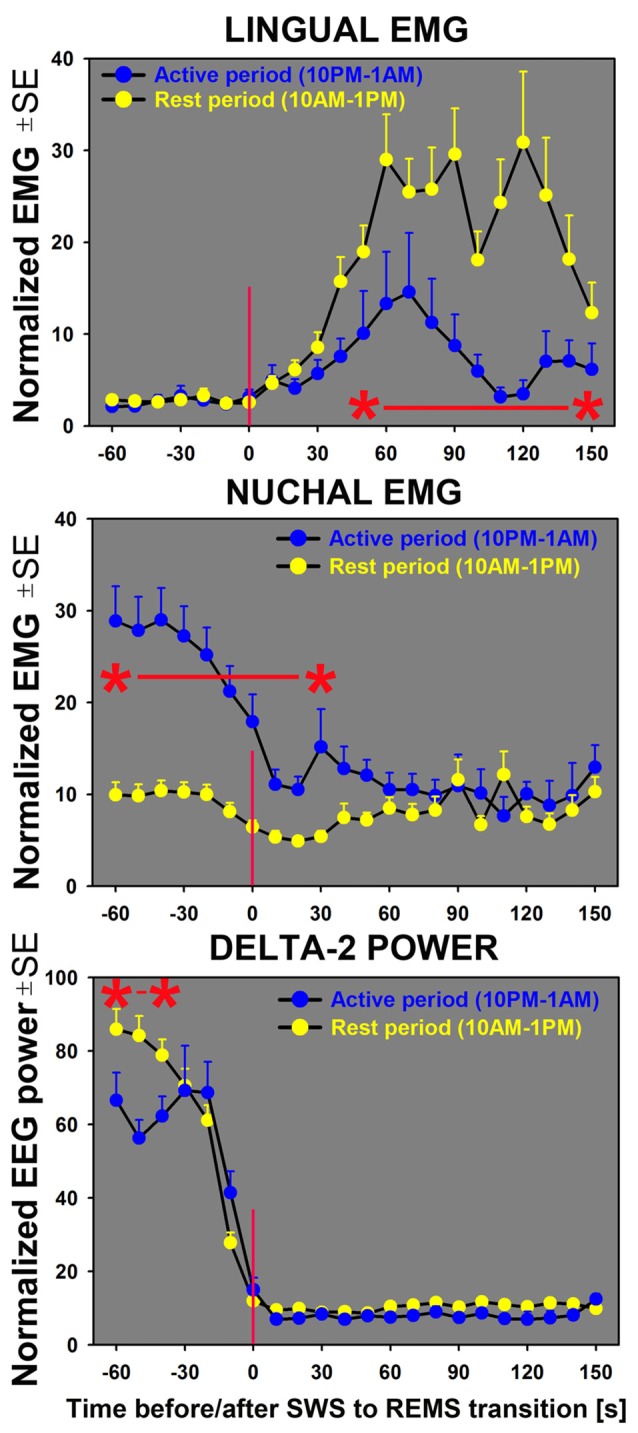
Sequential analysis of the time course of changes in lingual and nuchal EMGs during transitions from SWS to REMS indicates that lingual EMG (top graph) during REMS attains higher levels during the rest period (yellow symbols) than during the active period (blue symbols). For nuchal EMG (middle graph), the level of activity prior to the state transition is significantly higher during the night/active period that during the day/rest period. The bottom graph shows the time course of concurrently measured EEG delta-2 power (0.5–2.0 Hz). The sharp decline of the signal near zero time verifies the accuracy of state transition scoring; the significantly lower delta-2 power for the SWS episodes occurring during the first half of the active period (10:00 pm–1:00 am) than for the first half of the rest period (10:00 am–1:00 pm) reflects the well-established rest-activity rhythm of this signal (Grasing and Szeto, 1992; Ferri et al., 2001; Borbély et al., 2016). All curves represent average, normalized EMG and EEG signals concurrently measured in successive 10 s intervals during the SWS to REMS transitions in seven rats during 3 h of active period (*n* = 38) and 3 h of rest period (*n* = 42). Asterisks (*) and red horizontal lines mark the periods when the differences between the two circadian phases were significant at *p* < 0.05, as determined by unpaired *t*-tests.

Our analysis of the time course of lingual and nuchal EMGs during the period just preceding and following the transition from SWS to REMS extended the findings pertaining to the rest/active phase-dependance of lingual and nuchal EMGs during sleep states that emerged from the averaging across entire sleep periods (Figure 4). The top graph in Figure 5 presents a comparison of the average time course of lingual EMG measured during the successive 10 s-long epochs preceding and following transition from SWS to REMS obtained from the night/active period and day/rest period (the methods used to align and superimpose EMG levels during multiple SWS-REMS transitions in order to obtain a smoothed time-dependance of muscle twitches that characterize lingual EMG during REMS were described previously (Rukhadze et al., 2011). As documented in earlier studies limited to the rest period of circadian cycle (Lu et al., 2005; Lu and Kubin, 2009; Rukhadze et al., 2011, 2014), lingual EMG during REMS takes a form of semi-random twitches whose magnitude and frequency gradually increase with time after REMS onset. Our present analysis revealed that, from about 1 min into REMS episodes, the mean magnitude of lingual EMG attained a significantly higher level during the REMS periods occurring during the day/rest period than during the night/active period.

In contrast, the time course of concurrently measured nuchal EMG (Figure 5; middle graph) was opposite of that for lingual EMG. During both day and night periods, nuchal EMG gradually declined from a measurable level during SWS to atonia during REMS. However, the starting levels of nuchal EMG prior to the entry into REMS were significantly higher for the state transitions occurring during the night/active period than during the day/rest period.

### mRNA Expression Differences Between the Rest Onset and Active Period Onset in the XII Nucleus and Inferior Olive (IO)

Our transcriptional analysis of differences between the rest period onset and active period onset in the XII nucleus and IO revealed multiple transcripts that differed at high levels of statistical significance. The complete raw results from this part of this study are listed in the spreadsheet file associated with this report (Supplementary Material Table S1). Generally, more transcripts had significant differences for the samples from the IO than for those from the XII nucleus. This was likely related to the XII nucleus having a more homogenous cellular composition (mostly motoneurons and glial cells) than IO (note in Figure 1B that the tissue punches taken from the XII nucleus did not extend beyond the boundaries of the nucleus, whereas those taken from IO inevitably included cells from adjacent regions). With a relatively liberally set threshold for significance of day-night differences at 0.01, as many as 596 out of 14,708 known transcripts were different in the XII nucleus and IO combined. For the *p* value threshold of 0.001, there were 131 transcripts that exhibited a day-night difference. Of those, 25 differed significantly in both the XII nucleus and IO, with all but one (*Idi1*) showing the same day-night direction of change in both locations. Another 15 transcripts had a significant day-night difference in the XII nucleus only, and 91 had significant day-night difference in the IO only. In each of these subsets, there were transcripts representing the core molecular circadian oscillator, such as *Arntl/Bmal1*, *Clock*, *Csnk1e*, *Cry1*, *Cry2*, *Dbp*, *Errfi1*, *Npas2*, *Nr1d1/Rev-erbα*, *Per1*, *Per2*, *Per3*, *Rora* and *Tgfa*.

We focused on the clock-related transcripts and those for which the *p* value for their day-night difference in the XII nucleus was lower than 0.001. This data subset is listed in the last tab of the worksheet associated with this report, with key parts of that worksheet shown in Table 1. Additionally, Table 1 includes a comparison between the outcomes from our microarray study and the transcriptional circadian oscillations detected in the mouse brainstem published in “CIRCA” database (CircaDB, 2018).

**Table 1:** Comparison of transcriptional data in “CIRCA” database for the mouse brainstem (http://circadb.hogeneschlab.org/) and the day-night differences in the XII nucleus and inferior olive (IO) revealed by our microarray study.

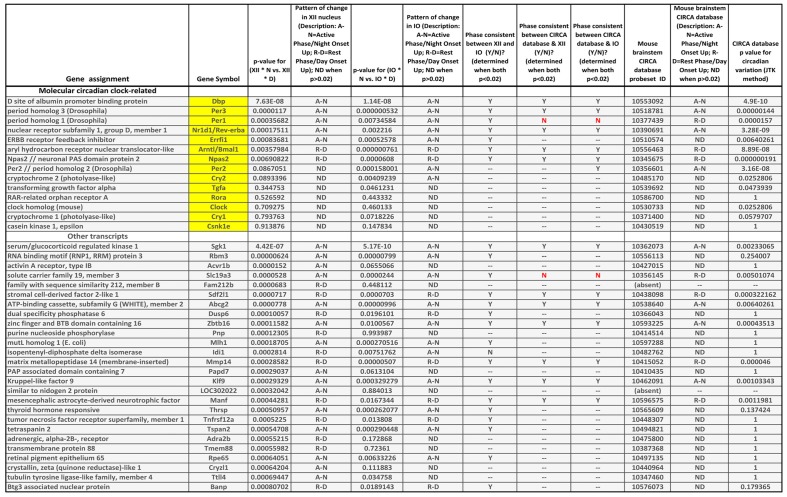

*The table separately lists major molecular circadian clock-related transcripts and other transcripts whose levels in the XII nucleus differed between the rest onset and active period onset at statistical significance levels of *p* < 0.001 (see text for details). Bold, red N marks the few transcripts for which the phase of circadian change differed between the whole mouse brainstem, as listed in “CIRCA” database, and in our tissue samples from either the XII nucleus or IO*.

The following are our most noteworthy findings. Of the 14 transcripts considered because they represented important parts of the molecular circadian oscillator, five had rest onset-active period onset differences in the XII nucleus that were significant at *p* levels less than 0.001 (*Dbp*, *Errfi1*, *Nr1d1/Rev-erbα*, *Per1* and *Per3*). The direction of the day-night difference for all these transcripts was the same in the XII nucleus and IO, and for only one transcript (*Per1*) the direction of day-night change was not the same as in “CIRCA” database for the mouse brainstem. Three additional circadian clock-related transcripts (*Arntl*, *Npas2* and *Per2*) had significant day-night differences at the cut off *p* value of 0.001 in the IO but not the XII nucleus.

The 26 transcripts other than those belonging to the molecular clock for which we detected significant day-night differences in the XII nucleus at *p* < 0.001 (Table 1) were of diverse types. Seven represented various enzymes (e.g., kinases, phosphatases or ligases), suggesting a metabolic component in the local rest-activity regulation in the motor nucleus. There was a transcript for a nuclear protein, another one for an RNA biding motif, and one for the adrenergic alpha-2b receptor. The latter may have been derived from local astrocytes because this receptor mRNA is absent from XII motoneurons (Volgin et al., 2001). Sixteen of the 26 transcripts had changes in the same direction in the IO, and seven of those were also listed in “CIRCA” database as significantly oscillating with circadian rhythm and a similar phase in the entire mouse brainstem. For only one transcripts showing a significant day-night difference in the XII nucleus, its direction of change was opposite in the XII nucleus and the mouse brainstem (*Slc19a3*), thus supporting the validity of our use of the significance level of *p* < 0.001 as a relevant cut-off for non-spurious effects.

## Discussion

Our main findings are that the effect of sleep on spontaneous lingual and nuchal EMG levels are not uniform and vary with both the circadian phase of the rest-activity cycle and sleep stages, and that associated with these EMG changes are transcriptional changes at the level of the XII motor nucleus and IO, two medullary nuclei of which one directly determines the motor output and the other is important for sensorimotor integration. These findings reveal that motor output from both spinal-postural and cranial-orofacial muscles is modulated in a manner that helps align these motor outputs with the circadian rhythm of the rest-activity cycle. In addition, in the case of lingual muscles, our findings are of potential clinical significance for a better understanding of the ability of these muscles to protect the upper airway against collapse in the OSA syndrome.

### Pattern of State-Dependent Changes in Lingual and Nuchal EMGs During the Rest and Active Phases of the Circadian Cycle

Rats are nocturnal animals and exhibit a strong circadian rhythmicity of locomotor activity and proportions of time spent in sleep and wakefulness. Concurrently with these overt behavioral expressions of circadian rhythm, EMG generated during bouts of wakefulness becomes gradually more intense over the duration of the active period parallel to the progressively increasing percentage amount of wakefulness. This pattern results from the interaction between the effects that emanate from the central circadian clock, commonly referred to as the “process C”, and the opposing effect of increasing sleepiness referred to as the “process S” (Borbély, 1982; Borbély et al., 2016). This two-process model has been well validated based on EEG changes across the rest and active phases of the circadian cycle. In the present study, we focused on quantification of EMG levels during wake, SWS and REMS states, as they occur across the entire circadian cycle and we found new features that could not be extrapolated without a thorough analysis across the entire 24 h period. Specifically, we found that the average lingual and nuchal EMGs during wake periods occurring during the rest phase of circadian cycle attain only about 60% of the levels during wakefulness occurring during the active phase of the circadian cycle. This difference is, at least in part, due to the difference in intensity of various consciously controlled behaviors during the day and night, but additional network, cellular, biochemical, neurochemical and molecular mechanisms may contribute at multiple premotor and motor levels to help maximize motor efforts consciously initiated during the active periods and suppress such efforts when they are generated during the rest period of circadian cycle.

An explanation of our findings solely based on differences in volitional control of motor behaviors is not sufficient to explain the differences in EMG levels between the rest and active phases of circadian cycle that we uncovered during either SWS or REMS. For nuchal EMG, its level during SWS occurring during the active phase of circadian cycle was about three times higher than during SWS occurring during the rest phase. This cannot be explained by volitional control and strongly suggests the presence of circadian phase-dependent mechanisms whose purpose would be to enhance postural activity during SWS at the time principally designated for execution of active behaviors and maintenance of attention to the environment. Such an enhanced nuchal tone may help maintain upright head position and facilitate orienting behaviors.

As with nuchal EMG, the relatively enhanced wake-related lingual activity during the night/active period when compared to the day/rest period is probably driven behaviorally and through specific circadian mechanisms designed to reinforce the behavioral activation. In contrast to nuchal EMG, we found that lingual EMG during REMS was relatively enhanced during the rest phase of circadian cycle when compared to the REMS episodes occurring during the active phase. In our prior studies in rats (Lu et al., 2005; Lu and Kubin, 2009; Rukhadze et al., 2011), we documented that lingual EMG during REMS is strictly phasic, with multiple twitches generated in a semi-random fashion by otherwise inactive muscles, and with the frequency and intensity of twitching gradually increasing after the REMS episode onset. A similar, non-respiratory twitching of lingual muscles during REMS has been described in healthy humans (Chokroverty, 1980). Accordingly, the difference in the mean level of lingual EMG during REMS between the rest and active periods is due to different intensities of lingual muscle twitches that characterize lingual EMG during REMS. Whether there is any distinct physiologic advantage in the enhanced occurrence of REMS-related twitches during the rest phase of circadian cycle is not clear. It may be of interest to determine whether other cranial muscles that exhibit intense bursts of phasic activity during REMS, such as oculomotor, trigeminal or laryngeal (Gadea-Ciria, 1976; Kuna et al., 1994; Fraigne and Orem, 2011; Kato et al., 2013), also generate more intense twitching during the REMS episodes produced during the rest phase of circadian cycle.

The differences in sleep state effects on nuchal and lingual EMG between the rest and active phases of circadian cycle strongly suggest that these two motor outputs have their distinct underlying mechanisms; such mechanisms may reside within the specialized premotor networks for these different motor outputs or right within their corresponding pools of motoneurons. The comparison of our microarrays data obtained from the XII motor nucleus and a site located considerable upstream from motoneurons (the IO) indicates that different sets of transcripts exhibit highly significant rest-active phase differences; this would be consistent with at least some aspects of the rest period-active period modulation of EMG levels in different sleep-wake states being exerted within individual motor nuclei.

To address one possible concern that the day-night differences between lingual and nuchal EMGs that we detected by comparing measures averaged across the entire durations of individual bouts of sleep and wake states could be secondary to day-night differences in individual sleep and wake episode durations, we quantified the time course of the lingual and nuchal EMG levels during fixed periods aligned to individual transitions from SWS to REMS. This analysis eliminated any potential bias related to the different durations of individual sleep bouts during the rest and active phases of circadian cycle and still indicated that the SWS-related nuchal EMG level was significantly elevated during the night/active period, whereas the REMS-related lingual EMG was significantly elevated during the day/rest period.

### Transcriptional Differences Between the Rest Period Onset and Active Period Onset in the XII Motor Nucleus, Inferior Olive (IO) and the Entire Mouse Brainstem

A comparison of our microarray data from the XII nucleus and IO to “CIRCA” database for the entire mouse brainstem (CircaDB, 2018) revealed considerable consistency among these three sites/tissues for many transcripts. Nevertheless, the agreement was far from complete, with many differences among the three sets of significantly changing transcripts. The extremely high levels of statistical significance for some of the transcripts suggest that those were real differences in transcriptional rhythms in the three locations. Due to the high diversity of the non-circadian clock transcripts with highly significant day-night differences, it would be speculative at this stage of our knowledge to discuss any potential functional implications but the presence of such a diversity points to the presence of a fine regional regulation. As a follow up on our findings, it would be important to determine which of these differences are driven by the central circadian clock(s) *per se*, which are secondary to the varying amounts of sleep between day and night, and which are activity- or use-dependent.

With the regard to molecular clock-related transcripts, a review of “CIRCA” database reveals that only three transcripts (*Dbp*, *Nr1d1/Rev-erbα* and *Per3*) consistently and robustly oscillate in the mouse brainstem, mouse hypothalamus, mouse suprachiasmatic nucleus and mouse liver, whereas other molecular clock transcripts listed in Table 1 that exhibited significantly changes in the XII nucleus do not necessarily change with circadian rhythm in all these brain locations and organs. This indicates that not all major molecular clock genes are necessary at each site to produce, or reinforce, local circadian rhythms. Accordingly, our findings suggest that *Dbp*, *Errfi1*, *Nr1d1/Rev-erbα*, *Per1* and *Per3* have a key role in circadian rhythmicity in the XII nucleus, whereas *Clock*, *Cry1*, *Cry2*, *Csnk1e*, *Per2*, *Rora* and *Tgfa* are relatively unimportant, and the roles of *Arntl/Bmal1* and *Npas2* are limited based on an intermediate statistical significance of their rest-activity changes (*p* = 0.0035–0.0069).

It remains to be determined whether the day-night differences in selected molecular clock and other transcripts that we identified in the XII nucleus and IO have an important role in locally adjusting cellular activity to best match the needs associated with the rest and active phases of circadian cycle. Our combination of functional measurements of EMG levels and local transcriptional activities make this highly plausible. Furthermore, in other brain regions and peripheral organs, local molecular clocks actively contribute to optimal alignment with the rest-activity rhythm of sensory processing in the cochlea and inferior colliculus, or metabolism in the liver (Albrecht, 2012; Musiek et al., 2013; Park et al., 2016).

### Conclusions and Implications for Motor Control in Humans, Including Upper Airway Control in OSA

When wakefulness occurs during the normal rest phase of circadian cycle, hence under the conditions of circadian misalignment, wake-related activity of lingual and nuchal muscles is lower compared to that generated during wakefulness occurring during the active phase of circadian cycle. The same pattern also holds for activity of dorsal neck (postural) muscles during SWS. For the rat lingual EMG quantified during SWS, this pattern may be obscured due to the “floor” effect because lingual EMG is atonic, or nearly atonic, during this phase of sleep (Lu et al., 2005; Lu and Kubin, 2009). In contrast, the rest-active phase difference for lingual EMG during REMS is definitely opposite of that during either wakefulness or during SWS for the nuchal EMG. Specifically, lingual EMG level during REMS is higher during the rest phase than during the active phase of circadian cycle.

Our EMG measurements during wakefulness indicate that, at least in rats, a misalignment of sleep relative to the circadian cycle may adversely influence execution of motor tasks. Although EMG measurements compatible to ours are not available for human subjects, indirect evidence suggests that circadian misalignment of sleep and wake states has a measurable impact also in humans. For example, circadian misalignment impedes the ability to achieve maximal performance in athletes (Thun et al., 2015), and impairs motor performance in persons whose baseline motor skills are compromised by aging or motor disability (Lauretti et al., 2017). One may also argue that the relatively higher level of nuchal EMG during SWS when it occurs during the active phase of circadian cycle, as well as the increased lingual EMG during REMS when it occurs during the rest phase of circadian cycle, may have a disruptive effect on sleep continuity and quality. This, in turn, may adversely affect the restorative functions of sleep.

With the regard to upper airway control in OSA, which is an almost uniquely human disorder, data suggest that the frequency and duration of breathing events and the critical closing pressure during SWS vary with ToD (El-Chami et al., 2015). An important background for the interpretation of these findings may be that, in contrast to rats and human subjects with fully patent upper airway, lingual EMG in OSA patients is not atonic during SWS (Katz and White, 2003). With some activity present, differences may also be present between SWS occurring during the rest and active phase of circadian cycle. During REMS, in turn, OSA patients who are concurrently affected by REMS behavior disorder which causes enhanced phasic twitching in multiple muscles during REMS (Frauscher et al., 2008) have less severe OSA during this stage of sleep than control OSA subjects (Huang et al., 2011). This would suggest that the relatively increased phasic lingual twitches during REMS when the state occurs during the rest phase of circadian cycle may protect against airway obstructions. These findings point to both usefulness and limitations of extrapolation of data from rats to OSA patients because their upper airway muscle control is different from healthy subjects.

## Author Contributions

KH conducted analysis of locomotor activity, researched comparisons between our microarray data and “CIRCA” database, assisted with instrumentation surgeries. GM conducted instrumentation surgeries, scored sleep-wake behavior, extracted and analyzed the time course of signals during SWS to REMS transitions. LK designed the study and guided data analysis, assisted with instrumentation surgeries, prepared the first draft of the manuscript.

## Conflict of Interest Statement

The authors declare that the research was conducted in the absence of any commercial or financial relationships that could be construed as a potential conflict of interest.
